# Associations between serum CA724 and HER2 overexpression among stage II–III resectable gastric cancer patients: an observational study

**DOI:** 10.18632/oncotarget.8145

**Published:** 2016-03-17

**Authors:** Xin-Zu Chen, Wei-Han Zhang, Hai-Ning Chen, Jian-Ping Liu, Du He, Yang Liu, Kai Liu, Xiao-Long Chen, Xian-Ming Mo, Zong-Guang Zhou, Jian-Kun Hu

**Affiliations:** ^1^ Department of Gastrointestinal Surgery, West China Hospital, Sichuan University, Chengdu, China; ^2^ Laboratory of Gastric Cancer, State Key Laboratory of Biotherapy/Collaborative Innovation Center of Biotherapy, West China Hospital, Sichuan University, Chengdu, China; ^3^ Department of Pathology, West China Hospital, Sichuan University, Chengdu, China; ^4^ West China School of Public Health, Sichuan University, Chengdu, China; ^5^ Laboratory of Stem Cell Biology, State Key Laboratory of Biotherapy, West China Hospital, Sichuan University, Chengdu, China

**Keywords:** gastric cancer, CA724, human epidermal growth factor receptor 2, immunohistochemistry, carbohydrate antigen

## Abstract

**Objectives:**

Associations between serum tumor biomarkers and human epidermal growth factor receptor 2 (HER2) overexpression among locally advanced gastric cancer patients were yet to be determined and therefore warranted investigation.

**Results:**

A total of 318 patients were analyzed. The odds ratios of CA724 were 4.79 (95% CI 1.55–14.79) and 6.29 (1.40–28.19) in comparing the HER2 (2+/3+) and HER2 (3+) with the negative group, respectively (*p* < 0.05). A combination of the four biomarkers yielded slightly but not significantly greater areas under the curve (AUC = 0.83; 0.71–0.94) than that of serum CA724 alone (0.80; 0.68–0.91); however, an index generated from the combination had better diagnostic performance with 85.7% sensitivity, 80.4% specificity and 97.8% negative predictive value to predict the strong overexpression of HER2 (3+). CA199, CEA or CA125 alone was not associated with HER2 overexpression. Leave-one-out cross-validation found a consistent association between serum CA724 and HER2 (2+/3+) overexpression.

**Methods:**

Patients undergoing radical gastrectomy from 8/2012 to 12/2013 and with pathological stage II–III gastric cancer were retrospectively analyzed. HER2 expression of the surgical samples was estimated using immunohistochemistry; serum CA724, CA199, CEA and CA125 were preoperatively tested. Internal validation was performed using the leave-one-out approach.

**Conclusions:**

Serum CA724 is significantly associated with the overexpression of HER2 among locally advanced gastric cancer patients. The combination of CA724, CA199, CEA and CA125 is better than serum CA724 alone in predicting HER2 overexpression. External validation and further investigation of the biological mechanisms of these associations are required.

## INTRODUCTION

Gastric cancer was the fifth most common cancer and the third most common cause of cancer-related death in the world in 2012 [1]. The proportion of locally advanced or metastatic diseases was no less than 80% of all gastric cancer patients in mainland China, [2, 3] and a multidisciplinary treatment including targeted therapy may play many roles in the management of gastric cancer. [4–6]. Human epidermal growth factor receptor 2 (HER2) has been recognized as a marker for targeting therapy with trastuzumab for metastatic gastric cancer [7]. The expression of HER2 is up-regulated in more than 20% of metastatic gastric cancer patients [8]. Moreover, the expression of HER2 among resectable gastric cancer patients is underestimated to some extent [9]. Whether the overexpression of HER2 in locally advanced gastric cancer is a prognostic factor or an indicator of neo-adjuvant therapy has been investigated by some researchers. Moreover, Berretta et al. first reported a case with the administration of intraperitoneal trastuzumab to treat peritoneal metastatic gastric cancer [10]. The overexpression of HER2 may also indicate the future potential need for neo-adjuvant or intraoperative trastuzumab therapy.

The definition of HER2 expression is based on the pathological examination of tissues, especially surgical samples [7]. Yoshida et al. found a concordance rate of immunohistochemistry (IHC) results between surgical specimens and the corresponding biopsies of up to 57% and a low kappa value of 0.224; [11]. however, Wang et al. found a strong concordance [12]. A possible reason is the intratumoral heterogeneity of HER2 expression in gastric cancers, and at least 4 biopsy tissues containing cancer cells are suggested [13]. Therefore, additional approaches that preoperatively and efficiently predict HER2 expression levels are warranted. It may be cost-effective to select high-risk individuals first through preoperative serological tests and to subsequently require the detection of HER2 expression in surgical specimens. Peng et al. and Zhou et al. found that the serum HER2 extracellular domain (ECD) was highly correlated with tissue HER2 status in metastatic gastric cancer; there was also a significant difference in the serum HER2 ECD levels between patients with HER2 IHC 3+ and those with HER2 IHC 2+/FISH+, which supported the clinical utility of serum HER2 ECD detection in patients with advanced gastric cancer [14, 15]. However, there are currently no serological approaches to predict the expression level of HER2 among locally advanced gastric cancer patients. Conventional serum tumor biomarkers, including CA724, CA199, CEA and CA125, are potentially associated with the detection and prognosis of gastric cancer [16]. Therefore, this study investigated the associations between the HER2 expression level and conventional serum tumor biomarkers and assessed their strength in predicting the overexpression of HER2 among locally advanced gastric cancer patients.

## RESULTS

### HER2 prevalence and patient characteristics

A total of 318 patients with stage II–III gastric cancer were analyzed. The prevalence of IHC HER2 (2+/3+) and (3+) were 20.1% (64/318) and 6.3% (20/318), respectively. The general clinicopathological characteristics are shown in Table 1. Age, gender, family history of malignancies, tumor size, tumor site, Borrmann type and TNM stage of the IHC HER2-negative group were all comparable with either the HER2 (3+) or (2+/3+) group (*p* > 0.05). Only the cases with moderately differentiated tumors had a higher prevalence in the IHC HER2 (3+) group (*p* = 0.01).

**Table 1:** Clinicopathological characteristics of studied patients

Characteristics	Negative	Overexpression			
	HER2 (0/1+) *N* = 254	HER2 (3+) only *N* = 20		HER2 (2+/3+) *N* = 64	
	*n* (%)	*n* (%)	p*	*n* (%)	p*
Age (years)^a^			0.28		0.67
< 40	18 (81.8)	3 (13.6)		4 (18.2)	
40–59	113 (78.5)	9 (6.3)		31 (21.5)	
60–79	115 (80.4)	8 (5.6)		28 (19.6)	
≥ 80	8 (88.9)	0		1 (11.1)	
Gender^b^			0.08		0.15
Male	175 (82.2)	10 (4.7)		38 (17.8)	
Female	79 (75.2)	10 (9.5)		26 (24.8)	
Family history of malignancies^c^	5 (83.3)	1 (16.7)	0.37	1 (16.7)	1.00
Tumor site^b^			0.36		0.99
U/UE	71 (78.0)	9 (9.9)		20 (22.0)	
UM/MU	6 (75.0)	0		2 (25.0)	
M	17 (81.0)	1 (4.8)		4 (19.0)	
LM/ML	10 (83.3)	2 (16.7)		2 (16.7)	
L/LD	141 (80.6)	7 (4.0)		34 (19.4)	
UML	9 (81.8)	1 (9.1)		2 (18.2)	
Tumor size^a^			0.45		0.77
≤ 3 cm	37 (80.4)	4 (8.7)		9 (19.6)	
> 3 cm, ≤ 5 cm	83 (78.3)	7 (6.6)		23 (21.7)	
> 5 cm	134 (80.7)	9 (5.4)		32 (19.3)	
Differentiation^a^ (missing = 2)			0.01		0.35
Moderate	19 (73.1)	5 (19.2)		7 (26.9)	
Poor/undifferentiated	234 (80.7)	14 (4.8)		56 (19.3)	
Borrmann type^a^ (missing = 4)			0.32		0.82
0/I/II	111 (81.6)	10 (7.4)		25 (18.4)	
III	106 (75.7)	10 (7.1)		34 (24.3)	
IV	33 (86.8)	0		5 (13.2)	
TNM stage^a^			0.08		0.88
II	78 (80.4)	10 (10.3)		19 (19.6)	
III	176 (79.6)	10 (4.5)		45 (20.4)	

a Wilcoxon test.

b Chi-square test.

c Fisher's exact test.

* The *p* values of comparisons to negative group IHC HER2 (0/1+).

### Serum tumor biomarkers and the risk of HER2 overexpression

**Figure 1:**
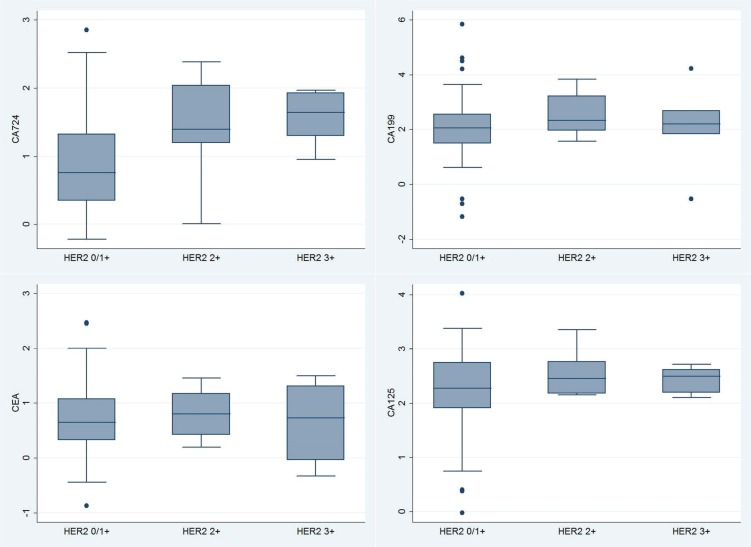
Box plots of Box-Cox transformed levels of serum CA724 (*p* = 0.017), CA199 (*p* = 0.421), CEA (*p* = 0.867) and CA125 (*p* = 0.493), compared among HER2 0/1+, 2+ and 3+ groups by the Kruskal-Wallis equality-of-populations rank test.

In comparing the level of serum biomarkers between the HER2 (0/1+), (2+) and (3+) groups, only serum CA724 was significantly higher in the HER (2+) and (3+) groups (Figure 1). In the multivariate logistic regression analysis, only serum CA724 was a significant correlative factor for HER2 overexpression in the HER2 (3+) group (OR = 6.29, 95% CI 1.40–28.19, *p* = 0.02) (Table 2). In contrast, serum CA199 (OR = 1.34, *p* = 0.40), CEA (OR = 0.34, *p* = 0.19) and CA125 (OR = 1.30, *p* = 0.78) had no significant associations with the overexpression of HER2; serum CEA in particular showed a trend toward an inverse association with the overexpression of HER2. Similar results were found in the comparison between the HER2 (2+/3+) and HER2 (0/1+) groups (Table 2), and serum CA724 was significantly associated with the expression of HER2 (2+/3+) (OR = 4.79, 95% CI 1.55–14.79, *p* < 0.01).

**Table 2:** Serum CA724 as a correlative factor of IHC HER2 overexpression

Biomarkers (transformed)	HER2 (3+) only		HER2 (2+/3+)	
	OR (95% CI)	p*	OR (95% CI)	p*
CA724	6.29 (1.40–28.19)	0.02	4.79 (1.55–14.79)	< 0.01
CA199	1.34 (0.68–2.61)	0.40	1.42 (0.82–2.44)	0.21
CEA	0.34 (0.07–1.69)	0.19	0.45 (0.14–1.49)	0.19
CA125	1.30 (0.21–7.99)	0.78	1.97 (0.49–7.86)	0.34

Logistic regression without selection procedure.

* Compared to negative group HER2 (0/1+).

### Prediction of HER2 overexpression using serum tumor biomarkers

**Figure 2:**
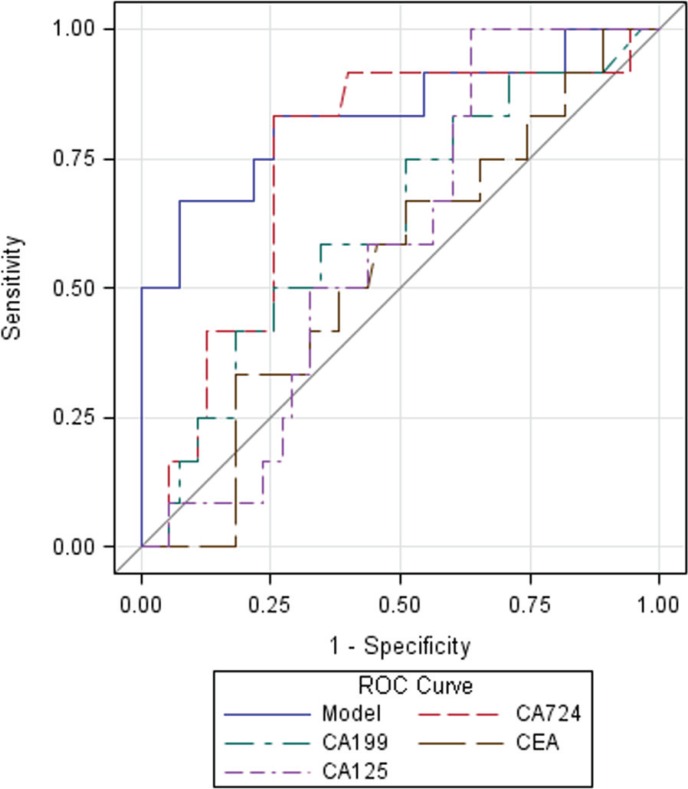
Receiver operating characteristic (ROC) curves of serum tumor biomarkers and their combination predicting HER2 overexpression

When comparing the HER2 (3+) subset with the HER2 (0/1+) subset, the AUC value of the four-biomarker combination was 0.83 (95% CI 0.71–0.94) (Figure 2). The AUC results did not significantly change (*p* > 0.05) when adjusting for eight covariates in either none selection or the stepwise backward selection model (Table 3). When comparing the combination with each serum tumor biomarker, only serum CA724 (AUC = 0.80, 95% CI 0.68–0.91, *p* = 0.71) was not inferior to the combination, whereas the AUCs of serum CA199, CEA and CA125 ranged from 0.51 to 0.54 (*p* < 0.01) (Table 3).

**Table 3:** Serum CA724 and combination predict IHC HER2 overexpression

Biomarkers (transformed)	HER2 (3+) only		HER2 (2+/3+)	
	AUC (95% CI)	p*	AUC (95% CI)	p*
Combination Model 1	0.83 (0.71–0.94)	Ref.	0.80 (0.68–0.92)	Ref.
Combination Model 2**	0.90 (0.79–1.00)	0.39	0.83 (0.68–0.99)	0.69
Combination Model 3***	0.79 (0.66–0.92)	0.67	0.77 (0.61–0.93)	0.82
CA724	0.80 (0.68–0.91)	0.71	0.75 (0.60–0.90)	0.63
CA199	0.53 (0.41–0.65)	< 0.01	0.52 (0.44–0.60)	< 0.01
CEA	0.54 (0.41–0.68)	< 0.01	0.50 (0.42–0.58)	< 0.01
CA125	0.51 (0.39–0.63)	< 0.01	0.49 (0.41–0.56)	< 0.01

Abbreviations: AUC, area under the curve; CI, confidence interval.

* Compared to combination Model 1 of CA724, CA199, CEA and CA125.

** Model 2 was Model 1 adjusted for age, gender, family history of malignancies, tumor site, tumor size, Borrmann type, differentiation grade, and TNM stage.

*** Model 3 was Model 2 by Logistic regression with stepwise backward selection procedure, while all other analyses were performed with none selection procedure.

Similar results could also be found in the comparison between the HER2 (2+/3+) and HER2 (0/1+) groups (Table 3). Namely, the four-biomarker combination (AUC = 0.80, 95% CI 0.68–0.92) predicted the expression of HER2 (2+/3+) significantly better than serum CA199, CEA or CA125 (*p* < 0.01), with the exception of serum CA724 (AUC = 0.75, 95% CI 0.60–0.90, *p* = 0.63).

### Diagnostic strength of serum tumor biomarkers in the HER2 (3+) subset

**Figure 3:**
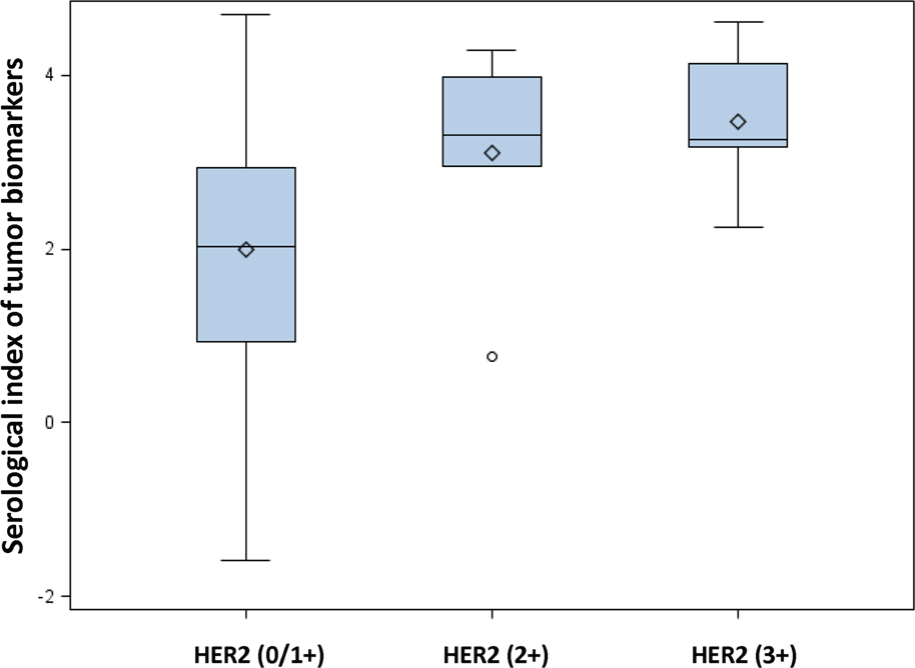
Box plot of serological indexes (SIs) among different classifications of HER2 expression

Based on the above logistic regression analysis in the HER2 (3+) subset, the SIs were calculated using the following format: SI = (CA724)_transformed_ × 1.84 + (CA199)_transformed_ × 0.29 – (CEA)_transformed_ × 1.09 + (CA125)_transformed_ × 0.26. The HER2 (2+) and HER2 (3+) subsets had higher SIs than that of the HER2 (0/1+) subset (namely HER2 (0/1+) vs. HER2 (2+), *p* = 0.05; and HER2 (0/1+) vs. HER2 (3+), *p* < 0.01) (Figure 3). As defined by the maximal Youden index, the optimal cutoff of the SI was > 2.252. Cases with SIs above the optimal cutoff were classified as positivity. The diagnostic parameters of SI and the four serum tumor biomarkers in detecting the overexpression of HER2 (3+) are shown in Table 4.

**Table 4:** Diagnostic performance in the strong overexpression subset of IHC HER2 (3+)

Predictors	SEN (95% CI)	SPE (95% CI)	PPV (95% CI)	NPV (95% CI)
SI*	85.7 (42.3–97.6)	80.4 (67.6–89.8)	35.3 (14.3–61.7)	97.8 (88.4–99.6)
CA724	57.1 (18.8–89.6)	74.6 (61.6–85.0)	21.1 (6.2–45.6)	93.6 (82.4–98.6)
CA199	15.8 (3.6–39.6)	78.1 (72.5–83.1)	5.3 (1.2–14.6)	92.3 (87.9–95.6)
CEA	25.0 (8.8–49.1)	74.3 (68.4–79.6)	7.3 (2.4–16.1)	92.5 (87.9–95.7)
CA125	15.8 (3.6–39.6)	91.7 (87.5–94.9)	13.0 (2.9–33.6)	93.3 (89.3–96.1)

Abbreviations: CI, confidence interval; NPV, negative predictive value; PPV, positive predictive value; SEN, sensitivity; SI, serological index; SPE, specificity.

* SI = (CA724)_*transformed*_ × β_*CA724*_ + (CA199)_*transformed*_ × β_*CA199*_ + (CEA)_*transformed*_ × β_*CEA*_ + (CA125)_*transformed*_ × β_*CA125*_

SI, and four serum tumor biomarkers performed well in the NPV analyses (all higher than 92%); however, all were weak in the PPV analyses with the best SI PPV of only 35.5% (95% CI 14.3%–61.7%) (Table 4). In analyses on the sensitivity, the SI had the highest SEN value (SEN = 85.7%, 95% CI 42.3%–97.6%), followed by serum CA724 (SEN = 57.1%, 95% CI 18.8%–89.6%). The specificity of the SI and the four serum tumor biomarkers were all above 74%. The specificity of the SI was 80.4% (95% CI 67.6%–89.8%), which was less than that of serum CA125 (SPE = 91.7%, 95% CI 87.5%–94.9%). The SI was the only one with both SEN and SPE higher than 80% in detecting HER2 (3+) expression.

### Leave-one-out cross-validation

Leave-one-out cross-validation was performed to compare the HER2 (2+/3+) subset to the HER2 (0/1+) subset. All individual results (Supplementary Table) were consistent with the primary analysis (Table 2). The combined results showed that serum CA724 was significantly associated with HER2 (2+/3+) overexpression (OR = 4.79, 95% CI 4.18–5.50).

## DISCUSSION

To our knowledge, this study is the first to report on the prediction of HER2 overexpression using conventional serum tumor biomarkers among locally advanced gastric cancer patients. This study found that the seropositivity of CA724 would be an independent correlative factor for HER2 overexpression. Likewise, serum CA724 had greater strength than CA199, CEA and CA125 in predicting the overexpression of HER2. The combination of CA724, CA199, CEA and CA125 had better sensitivity, specificity, and negative predictive value than those of serum CA724 alone in detecting HER2 overexpression.

First, this study found no associations between HER2 expression and the clinicopathological characteristics of locally advanced (stage II–III) gastric cancer patients. Gürel et al. also found no significant association between HER2 overexpression and tumor pathological characteristics in gastric cancer surgical specimens [17]. However, the finding of no association remains controversial and inconclusive. He et al. reported that HER2 overexpression was associated with Laurén classification and differentiation grade [18]. Movagharnejad et al. found that HER2 overexpression was greater in the intestinal type than the diffuse type but not associated with the degree of differentiation, tumor type, age, etc [19]. These findings partially address the difficulty in preoperatively predicating HER2 overexpression using only common clinicopathological characteristics.

Histological HER2 is a currently validated predictive biomarker for gastric cancer in trastuzumab target therapy [20]. The serum tumor biomarkers CA724, CA199, CEA and CA125 are associated with gastrointestinal cancers [21]. Knowledge of how to use these conventional serum tumor biomarkers to predict HER2 expression in gastric cancer is sparse. HER2 has potential predictive ability to estimate overall survival and may be a prognostic factor for gastric cancer. [22–24] Moreover, the serum tumor biomarkers CA724, CA199 and CEA are also associated with the prognosis of gastric cancer and likely as independent prognostic factors [25, 26]. Therefore, the rationale for this investigation is based on an association between HER2 and serum tumor biomarkers.

Tumor biomarker CA724 (also known as Tumor-associated glycoprotein 72, TAG-72) was suggested for the management of gastrointestinal and gynecological cancers as early as two decades previously; [27–29] however, the reason for a potential association between serum biomarker CA724 and HER2 overexpression is interesting but lacks relevant investigations. HER2 and TAG-72 are both considered excellent molecular targets for cancer imaging and therapy [30, 31]. Sharifzadeh et al. found that it was possible to develop oligoclonal nanobodies that target TAG-72 but that do no cross-react with HER2.30 Milenic et al. found that the dual targeting of 2 distinct molecules in tumors containing TAG-72 and HER2 with alpha particle radiation could result in an enhanced and additive therapeutic benefit in an animal tumor xenograft model [32]. The rationale for administrating monoclonal antibody mixtures is to overcome the heterogeneous nature of tumors in targeted therapy [32, 33]. We do not currently understand the potential biological mechanism for the association between CA724 and HER2; however, we can investigate their synergic function to improve the therapeutic effect.

Previous studies showed that serum CA724 performed substantially better than other serum tumor biomarkers in predicting gastric cancer [16, 21]. Likewise, in this study, only serum CA724 independently predicted the overexpression of HER2 among locally advanced gastric cancer patients. Therefore, this finding is consistent with the known feature of serum CA724 and suggests serum CA724 as a preferable serum biomarker for both gastric cancer and its overexpression of HER2. Furthermore, although the combination of CA724, CA199, CEA and CA125 did not lead to significantly greater AUCs than those of serum CA724 alone, the index generated from the combination had better diagnostic performance (85.7% sensitivity, 80.4% specificity and 97.8% negative predictive value) in predicting strong HER2 overexpression. Therefore, examination in a combination maneuver can be recommended in general practice. However, a shortcoming of this approach that warrants careful discussion is the relatively low positive predictive values of either the index examination or any of the individual biomarkers. Theoretically, the positive predictive value is also dependent on prevalence [34, 35]. The relatively low prevalence of HER2 (3+) in only 6.3% of these observations may partially contribute to the low positive predictive values of the biomarkers. Despite this, an index of the combination can further improve the positive predictive value from 21.1% of CA724 alone to 35.3%. Thus, the multiplex examination of CA724, CA199, CEA and CA125 is not adequate in predicting the overexpression of HER2 among locally advanced gastric cancer patients; however, the combination of preoperative IHC of the biopsy and serum biomarker multiplex could be used to obtain a promising predictive value.

Additionally, several limitations in this study warrant discussion. First, the proportion of IHC HER2 (3+) patients was a small group in the present series (6.3%, 20/318). In multicenter, cross-sectional studies based on Chinese gastric cancer patients, the positivity of HER2 was 12%–13% in IHC and ISH tests [5, 36]. Therefore, the present sample size with a small group of HER2 overexpression was unable to eliminate the type II error. Second, in this study, the FISH test was not preformed to explicitly classify the HER2 (2+) cases as overexpressed or not. Strictly speaking, FISH (or Dual ISH) needed to be performed in the cases with HER2 (2+) to precisely select true positive cases of HER2 overexpression. [37–39] Therefore, only considering HER2 (3+) as having strong overexpression should make the overexpression rate be underestimated, whereas the combination of HER2 (2+) and (3+) must overestimate the overexpression rate. In spite of that, the additional combination of HER2 (2+) and (3+) can be considered as an approach for sensitivity analysis. The analysis on HER2 (3+) only or a HER2 (2+/3+) combination demonstrated generally similar results, which indicated that the absence of a FISH test may not significantly influence the conclusions. Third, the individual serological biomarker tests were not performed in all identified observations, namely, the combination of CA724, CA199, CEA and CA125 among only 69 patients. This may introduce a certain amount of sampling error into the results. Although leave-one-out cross-validation was involved in this analysis and led to consistent results, the robustness could be improved through external validation. Forth, the data for HER2 status of the preoperative biopsy specimens are not available in this case series because the HER2 IHC test has not been involved in routine practice at our hospital. Thus, the present study is unable to show a concordance between the preoperative and postoperative HER2 status; further investigations are required.

In conclusion, serum CA724 is significantly associated with the overexpression of HER2 among locally advanced gastric cancer patients; however, CA199, CEA and CA125 are not. The combination of CA724, CA199, CEA and CA125 is better than serum CA724 alone in predicting the overexpression of HER2. Serum biomarkers may efficiently predict the risk of HER2 overexpression in gastric cancer preoperatively as a Supplementary approach. External validation and further investigations into the biological mechanism of the associations between serum CA724 and HER2 immunohistochemical overexpression are required.

## MATERIALS AND METHODS

### Ethics

This study was based on a retrospective collection of surgical patients' medical records and used preoperative serological and postoperative pathological results. This study was approved by the Biomedical Ethical Committee of West China Hospital, Sichuan University. The participants did not give written informed consent due to the nature of the retrospective study; however, the patients' records were anonymized and de-identified prior to analyses by researchers. Other researchers in this study did not have access to the patients' identifying information or records prior to anonymization. The study complied with the World Medical Association Declaration of Helsinki regarding the ethical conduct of research involving human subjects.

### Patients

The patients who underwent radical gastrectomy from 8/2012 to 12/2013 were retrospectively collected from the Gastric Cancer Patient Registry Database in West China Hospital [40–42]. The eligible patients were proven to have stage II and III gastric cancer via postoperative pathological examination. All patients were neo-adjuvant chemotherapy- and/or radiotherapy-naïve. Basic patient information was retrieved including age, gender, and a family history of malignancies (first degree relatives). The histological and pathological characteristics were analyzed for tumor site, tumor size, Borrmann type, differentiation grade, and TNM stage. The patients with IHC information and serology results were eligible.

### Pathology

The postoperative pathological assessment was performed in a peer review manner by two independent pathologists in the Department of Pathology, West China Hospital. The surgical samples were 10% neutral formalin-fixed for 8 hours and then dehydrated. The paraffin-embedded blocks were prepared in sections. Hematoxylin and eosin staining was used to evaluate tumor differentiation, infiltration depth, and lymph node metastasis. The pathological staging was according to the AJCC 7th TNM system [43].

### Immunohistochemistry

The HER2 antigen was semi-quantitatively detected in sections of formalin-fixed, paraffin-embedded neoplastic tissue using an automated IHC slide staining device (Ventana Medical Systems, Inc.) according to manufacturer's instructions. Deparaffinization and rehydration were performed, and antigenic recovery was performed in buffer (pH 9.0, EDTA, 100°C, 40 min). After antigen retrieval, peroxidase was blocked to avoid endogenous peroxidase activity. Staining with DAB chromogen followed by counterstaining with hematoxylin was performed.

**Figure 4:**
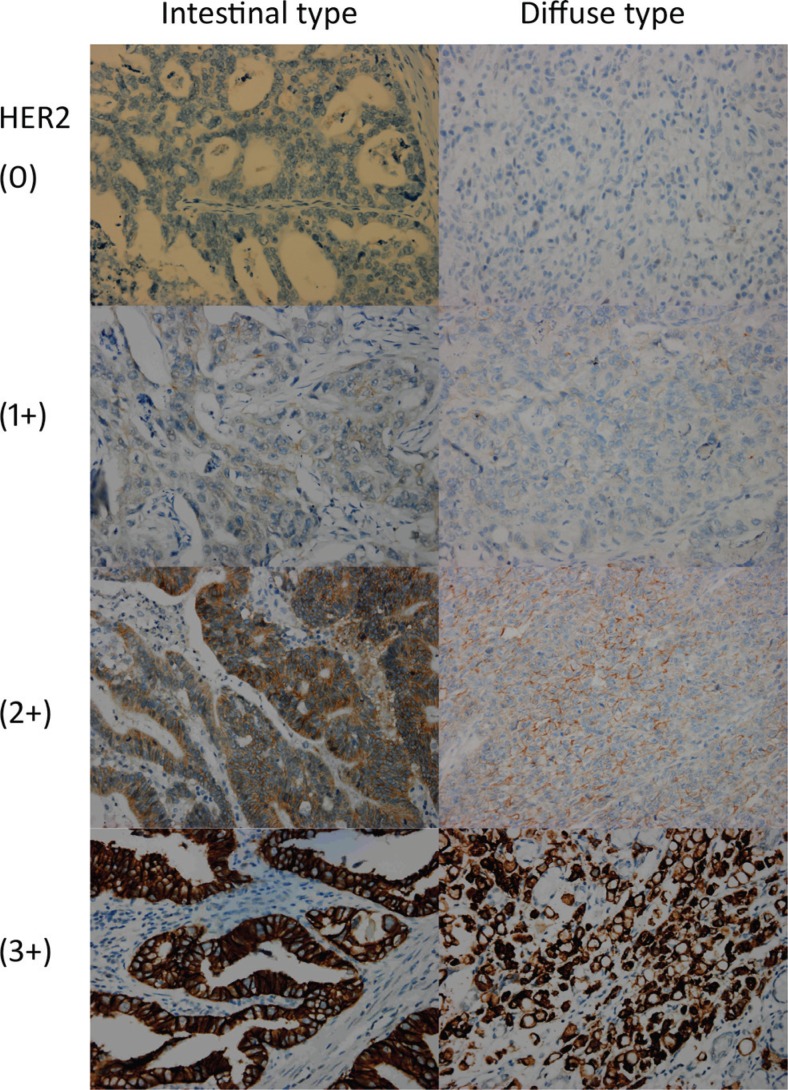
HER2 immunohistochemistry of gastric cancer tissues stratified by scores and Laurén classification

The scoring criteria were “0” for the cases without tumor cells or membrane stained, “1+” for weak and diffuse membrane staining of tumor cell clusters (> 10%), “2+” for lateral or basolateral complete but weak to moderate membrane staining of tumor cell clusters (> 10%) and “3+” for lateral or basolateral complete but strong membrane staining of tumor cell clusters (> 10%) (Figure 4). Subjects with a score of 0 or 1+ were defined has having negative expression; those with a 3+ score had definitive over-expression of HER2. Therefore, the subjects with 2+ or 3+ scores were combined and analyzed together as sensitivity analysis [44].

### Serological examination

Fasting peripheral venous blood was collected preoperatively in eligible patients. Fresh serum (200 μL) was tested using an Elecsys-2010 system (Roche Inc.) according to the manufacturer's instructions. The concentrations of tumor biomarker CA724, CA199, CEA and/or CA125 were determined preoperatively.

### Statistical analysis

To analyze baseline characteristics, the ranked variables were compared using a Wilcoxon test; the categorical variables were compared with Pearson's Chi-square test. The Kruskal-Wallis equality-of-populations rank test was used for nonparametric comparisons in multiple groups.

Because all of the serum levels for four biomarkers conformed to a positively skewed distribution in this study population, a linear transformation model (Box-Cox transformation) was performed for all variables of the four tumor biomarkers. The lambda values of the serum CA724, CA199, CEA and CA125 for the present observations were −0.28, −0.05, −0.3 and −0.05, respectively. Therefore, the transformed variables were calculated as (variable)_*transformed*_ = ((variable)_*original*_ (lambda)_*variable*_ – 1)/(lambda)_*variable*_.

The odds ratios (ORs) and 95% confidence intervals (CIs) were calculated via logistic regression based on the transformed continuous variables of biomarkers without a selection procedure. A receiver operating characteristic (ROC) curve was used to calculate area under the curve (AUC) of each serum tumor biomarker with standard error and 95% confidence interval (CI), respectively. The AUC value of the combination of four tumor biomarkers was regarded as a reference, and the comparisons of AUC values were performed using *Z* tests.

A serum index (SI) of four biomarkers was calculated as SI = (CA724)_*transformed*_ × β_*CA724*_ + (CA199)_*transformed*_ × β_*CA199*_ + (CEA)_*transformed*_ × β_*CEA*_ + (CA125)_*transformed*_ × β_*CA125*_. The value of β_i_ was the coefficient from the logistic regression. The optimal SI cutoff was defined as the value at the maximal Youden index (= sensitivity + specificity – 1). The sensitivity (SEN), specificity (SPE), positive and negative predictive values (PPV and NPV) of the SI and four biomarkers were calculated, respectively.

Due to a relatively low overexpression rate of HER2 in gastric cancer tissues, a leave-one-out cross-validation was performed as the internal validation. External validation was unavailable in the present study. The leave-one-out results were combined with a meta-analysis to confirm the associations between HER2 overexpression and serum biomarkers.

Two-sided *p* values of less than 0.05 were considered significant. The SAS 9.2 software, the STATA/SE 12.0 software and Comprehensive Meta Analysis Version 3.3.070 were used for statistical analysis where applicable.

### Compliance with ethical standards

All procedures performed in studies involving human participants were in accordance with the ethical standards of the Biomedical Ethical Committee of West China Hospital and with the 1964 Helsinki declaration and its later amendments or comparable ethical standards.

## SUPPLEMENTARY MATERIALS TABLE


