# Supplementary material for: Associations between serum CA724 and HER2 overexpression among stage II–III resectable gastric cancer patients: an observational study
Source: Oncotarget. 2016 Mar 17;7(17):23647–57. doi: 10.18632/oncotarget.8145 (PMC5029653; doi:10.18632/oncotarget.8145)
Supplement: Supplementary file 1 [file oncotarget-07-23647-s001.pdf]

# Associations between serum CA724 and HER2 overexpression among stage II–III resectable gastric cancer patients: an observational study

## Supplementary Materials

**Supplementary Table 1: Leave-one-out cross validation analyses comparing the HER2 (2+/3+) subset to the HER2 (0/1+) subset**

| Case left | Resampling | Serum CA724 (transformed) | | | Serum CA199 (transformed) | | | Serum CEA (transformed) | | | Serum CA125 (transformed) | | |
|---|---|---|---|---|---|---|---|---|---|---|---|---|---|
| | | OR | Lower 95% CI | Upper 95% CI | OR | Lower 95% CI | Upper 95% CI | OR | Lower 95% CI | Upper 95% CI | OR | Lower 95% CI | Upper 95% CI |
| 1 | 68 | 4.6903 | 1.4641 | 15.0259 | 1.4163 | 0.8212 | 2.4425 | 0.3797 | 0.1053 | 1.3695 | 2.1876 | 0.5117 | 9.3518 |
| 2 | 68 | 4.8325 | 1.5760 | 14.8181 | 1.5563 | 0.8529 | 2.8398 | 0.4882 | 0.1483 | 1.6075 | 1.9201 | 0.4926 | 7.4845 |
| 3 | 68 | 5.1263 | 1.6312 | 16.1104 | 1.4180 | 0.8267 | 2.4320 | 0.4500 | 0.1382 | 1.4658 | 2.0101 | 0.5027 | 8.0381 |
| 4 | 68 | 4.5803 | 1.4289 | 14.6821 | 1.7427 | 0.9121 | 3.3295 | 0.3707 | 0.0979 | 1.4033 | 1.6554 | 0.3903 | 7.0219 |
| 5 | 68 | 4.6722 | 1.5183 | 14.3775 | 1.4158 | 0.8226 | 2.4368 | 0.4519 | 0.1381 | 1.4786 | 1.9986 | 0.5043 | 7.9210 |
| 6 | 68 | 4.7282 | 1.5214 | 14.6945 | 1.4125 | 0.8192 | 2.4353 | 0.4552 | 0.1379 | 1.5025 | 1.9577 | 0.4920 | 7.7896 |
| 7 | 68 | 4.7034 | 1.5285 | 14.4731 | 1.4184 | 0.8219 | 2.4479 | 0.4407 | 0.1324 | 1.4667 | 1.8894 | 0.4675 | 7.6362 |
| 8 | 68 | 4.7034 | 1.5203 | 14.5506 | 1.4115 | 0.8192 | 2.4322 | 0.4540 | 0.1383 | 1.4908 | 1.9388 | 0.4872 | 7.7152 |
| 9 | 68 | 4.3555 | 1.4103 | 13.4508 | 1.3435 | 0.7700 | 2.3441 | 0.4325 | 0.1284 | 1.4570 | 1.7530 | 0.4337 | 7.0855 |
| 10 | 68 | 4.6746 | 1.5194 | 14.3821 | 1.4079 | 0.8187 | 2.4212 | 0.4551 | 0.1394 | 1.4859 | 2.0250 | 0.5083 | 8.0671 |
| 11 | 68 | 4.7107 | 1.5202 | 14.5975 | 1.4048 | 0.8129 | 2.4278 | 0.4583 | 0.1389 | 1.5130 | 1.9669 | 0.4961 | 7.7985 |
| 12 | 68 | 4.6802 | 1.5062 | 14.5422 | 1.4199 | 0.8236 | 2.4481 | 0.4552 | 0.1385 | 1.4963 | 1.9506 | 0.4921 | 7.7320 |
| 13 | 68 | 4.7091 | 1.5353 | 14.4439 | 1.4204 | 0.8255 | 2.4438 | 0.4509 | 0.1380 | 1.4728 | 2.0427 | 0.5121 | 8.1477 |
| 14 | 68 | 4.6845 | 1.5232 | 14.4068 | 1.4115 | 0.8205 | 2.4282 | 0.4569 | 0.1396 | 1.4950 | 1.9618 | 0.4970 | 7.7444 |
| 15 | 68 | 4.6879 | 1.5171 | 14.4857 | 1.4167 | 0.8216 | 2.4428 | 0.4460 | 0.1352 | 1.4713 | 1.9362 | 0.4869 | 7.7001 |
| 16 | 68 | 4.6511 | 1.5095 | 14.3312 | 1.4273 | 0.8272 | 2.4627 | 0.4524 | 0.1382 | 1.4811 | 2.0066 | 0.5062 | 7.9544 |
| 17 | 68 | 4.6816 | 1.5170 | 14.4476 | 1.4133 | 0.8214 | 2.4317 | 0.4601 | 0.1399 | 1.5129 | 1.9461 | 0.4924 | 7.6922 |
| 18 | 68 | 4.7043 | 1.5173 | 14.5856 | 1.4144 | 0.8212 | 2.4361 | 0.4575 | 0.1387 | 1.5093 | 1.9376 | 0.4862 | 7.7207 |
| 19 | 68 | 4.7415 | 1.4861 | 15.1281 | 1.4521 | 0.8310 | 2.5375 | 0.4760 | 0.1412 | 1.6046 | 2.2478 | 0.5337 | 9.4677 |
| 20 | 68 | 4.7278 | 1.5245 | 14.6619 | 1.4115 | 0.8183 | 2.4344 | 0.4539 | 0.1379 | 1.4940 | 1.9467 | 0.4877 | 7.7701 |
| 21 | 68 | 4.6869 | 1.5301 | 14.3565 | 1.3507 | 0.7555 | 2.4146 | 0.5055 | 0.1391 | 1.8373 | 2.0930 | 0.5150 | 8.5071 |
| 22 | 68 | 4.7086 | 1.5303 | 14.4879 | 1.4143 | 0.8203 | 2.4384 | 0.4447 | 0.1346 | 1.4694 | 1.8930 | 0.4687 | 7.6451 |
| 23 | 68 | 4.6821 | 1.5096 | 14.5218 | 1.4143 | 0.8214 | 2.4351 | 0.4570 | 0.1390 | 1.5024 | 1.9625 | 0.4959 | 7.7672 |
| 24 | 68 | 5.0152 | 1.6065 | 15.6570 | 1.4067 | 0.8190 | 2.4160 | 0.4321 | 0.1310 | 1.4254 | 2.0806 | 0.5144 | 8.4149 |
| 25 | 68 | 4.9204 | 1.5256 | 15.8692 | 1.4360 | 0.8216 | 2.5096 | 0.4526 | 0.1329 | 1.5418 | 2.2356 | 0.5245 | 9.5300 |
| 26 | 68 | 4.7266 | 1.5226 | 14.6726 | 1.4115 | 0.8182 | 2.4349 | 0.4526 | 0.1377 | 1.4883 | 1.9548 | 0.4909 | 7.7844 |
| 27 | 68 | 4.7872 | 1.5647 | 14.6461 | 1.4040 | 0.8164 | 2.4145 | 0.4519 | 0.1383 | 1.4766 | 1.8712 | 0.4687 | 7.4698 |

| | | | | | | | | | | | | | |
|---|---|---|---|---|---|---|---|---|---|---|---|---|---|
| 28 | 68 | 4.7162 | 1.5256 | 14.5801 | 1.4178 | 0.8228 | 2.4433 | 0.4577 | 0.1385 | 1.5127 | 1.8914 | 0.4568 | 7.8304 |
| 29 | 68 | 4.7300 | 1.5214 | 14.7053 | 1.4155 | 0.8213 | 2.4396 | 0.4541 | 0.1377 | 1.4970 | 1.9489 | 0.4879 | 7.7853 |
| 30 | 68 | 5.0478 | 1.6113 | 15.8139 | 1.4374 | 0.8335 | 2.4791 | 0.4316 | 0.1305 | 1.4270 | 2.1083 | 0.5190 | 8.5650 |
| 31 | 68 | 4.7788 | 1.5435 | 14.7954 | 1.4155 | 0.8206 | 2.4417 | 0.4518 | 0.1369 | 1.4913 | 1.9617 | 0.4892 | 7.8668 |
| 32 | 68 | 4.7405 | 1.5480 | 14.5164 | 1.4246 | 0.8270 | 2.4540 | 0.4500 | 0.1375 | 1.4731 | 1.9515 | 0.4942 | 7.7060 |
| 33 | 68 | 4.7409 | 1.5273 | 14.7166 | 1.4144 | 0.8204 | 2.4384 | 0.4534 | 0.1376 | 1.4940 | 1.9460 | 0.4853 | 7.8042 |
| 34 | 68 | 4.6934 | 1.5228 | 14.4655 | 1.4021 | 0.8129 | 2.4183 | 0.4539 | 0.1387 | 1.4851 | 1.9737 | 0.4985 | 7.8136 |
| 35 | 68 | 4.5916 | 1.4862 | 14.1859 | 1.3980 | 0.8166 | 2.3934 | 0.4831 | 0.1466 | 1.5923 | 2.3207 | 0.5027 | 10.7132 |
| 36 | 68 | 5.5191 | 1.6322 | 18.6621 | 1.4613 | 0.8213 | 2.6002 | 0.3604 | 0.0975 | 1.3325 | 2.1948 | 0.4902 | 9.8265 |
| 37 | 68 | 4.7561 | 1.5340 | 14.7463 | 1.4114 | 0.8164 | 2.4398 | 0.4536 | 0.1374 | 1.4978 | 1.9583 | 0.4905 | 7.8184 |
| 38 | 68 | 4.6912 | 1.5090 | 14.5840 | 1.4130 | 0.8208 | 2.4324 | 0.4613 | 0.1388 | 1.5326 | 1.9640 | 0.4962 | 7.7736 |
| 39 | 68 | 4.6635 | 1.5135 | 14.3699 | 1.4322 | 0.8262 | 2.4827 | 0.4181 | 0.1211 | 1.4441 | 2.0872 | 0.5123 | 8.5032 |
| 40 | 68 | 4.7057 | 1.5298 | 14.4746 | 1.4122 | 0.8197 | 2.4330 | 0.4498 | 0.1370 | 1.4770 | 1.9048 | 0.4755 | 7.6310 |
| 41 | 68 | 4.6303 | 1.5094 | 14.2039 | 1.4424 | 0.8352 | 2.4910 | 0.4844 | 0.1457 | 1.6100 | 2.0512 | 0.5188 | 8.1101 |
| 42 | 68 | 4.6298 | 1.4876 | 14.4096 | 1.3534 | 0.7864 | 2.3290 | 0.5148 | 0.1560 | 1.6991 | 1.9283 | 0.4796 | 7.7537 |
| 43 | 68 | 4.1737 | 1.3490 | 12.9131 | 1.3721 | 0.8002 | 2.3529 | 0.5593 | 0.1641 | 1.9064 | 1.8909 | 0.4904 | 7.2907 |
| 44 | 68 | 4.1864 | 1.3404 | 13.0755 | 1.4293 | 0.8181 | 2.4974 | 0.4420 | 0.1311 | 1.4906 | 1.9420 | 0.4867 | 7.7484 |
| 45 | 68 | 4.7251 | 1.5424 | 14.4754 | 1.4104 | 0.8197 | 2.4268 | 0.4501 | 0.1375 | 1.4734 | 1.9301 | 0.4878 | 7.6380 |
| 46 | 68 | 4.7543 | 1.5360 | 14.7160 | 1.4142 | 0.8202 | 2.4384 | 0.4541 | 0.1374 | 1.5004 | 1.9287 | 0.4687 | 7.9374 |
| 47 | 68 | 4.6411 | 1.5004 | 14.3558 | 1.4100 | 0.8201 | 2.4243 | 0.4582 | 0.1402 | 1.4970 | 2.0679 | 0.5100 | 8.3845 |
| 48 | 68 | 4.6907 | 1.5234 | 14.4435 | 1.4145 | 0.8216 | 2.4352 | 0.4553 | 0.1389 | 1.4926 | 1.9250 | 0.4849 | 7.6424 |
| 49 | 68 | 5.8850 | 1.7220 | 20.1126 | 1.4034 | 0.8197 | 2.4029 | 0.4072 | 0.1218 | 1.3613 | 2.7267 | 0.5910 | 12.5799 |
| 50 | 68 | 4.7967 | 1.5747 | 14.6114 | 1.3788 | 0.8040 | 2.3645 | 0.5095 | 0.1481 | 1.7525 | 1.9141 | 0.4914 | 7.4552 |
| 51 | 68 | 4.7967 | 1.5295 | 15.0433 | 1.2557 | 0.7275 | 2.1674 | 0.5216 | 0.1602 | 1.6982 | 1.9987 | 0.4916 | 8.1264 |
| 52 | 68 | 5.3303 | 1.6538 | 17.1806 | 1.4347 | 0.8322 | 2.4734 | 0.4141 | 0.1234 | 1.3897 | 2.2115 | 0.5298 | 9.2308 |
| 53 | 68 | 4.7204 | 1.5222 | 14.6385 | 1.4105 | 0.8177 | 2.4330 | 0.4536 | 0.1380 | 1.4913 | 1.9509 | 0.4901 | 7.7660 |
| 54 | 68 | 4.8536 | 1.5792 | 14.9167 | 1.3491 | 0.7675 | 2.3716 | 0.4491 | 0.1384 | 1.4574 | 1.9805 | 0.4993 | 7.8557 |
| 55 | 68 | 4.3010 | 1.3904 | 13.3039 | 1.3729 | 0.7939 | 2.3741 | 0.4785 | 0.1448 | 1.5815 | 1.9055 | 0.4810 | 7.5478 |
| 56 | 68 | 4.4994 | 1.4399 | 14.0593 | 1.3951 | 0.8109 | 2.4004 | 0.5426 | 0.1643 | 1.7926 | 2.2011 | 0.5402 | 8.9689 |
| 57 | 68 | 4.6513 | 1.4919 | 14.5013 | 1.4157 | 0.8222 | 2.4378 | 0.4548 | 0.1387 | 1.4911 | 2.0182 | 0.5035 | 8.0904 |
| 58 | 68 | 4.7561 | 1.5350 | 14.7364 | 1.4144 | 0.8203 | 2.4390 | 0.4535 | 0.1373 | 1.4977 | 1.9400 | 0.4776 | 7.8811 |
| 59 | 68 | 4.7274 | 1.5433 | 14.4809 | 1.4130 | 0.8208 | 2.4325 | 0.4476 | 0.1364 | 1.4685 | 1.9109 | 0.4813 | 7.5870 |
| 60 | 68 | 4.9638 | 1.5997 | 15.4029 | 1.4380 | 0.8312 | 2.4880 | 0.4233 | 0.1261 | 1.4211 | 1.9219 | 0.4812 | 7.6761 |
| 61 | 68 | 4.7625 | 1.5368 | 14.7589 | 1.4125 | 0.8175 | 2.4405 | 0.4530 | 0.1372 | 1.4949 | 1.9568 | 0.4889 | 7.8312 |

| | | | | | | | | | | | | | |
|---|---|---|---|---|---|---|---|---|---|---|---|---|---|
| 62 | 68 | 5.7855 | 1.7131 | 19.5386 | 1.2990 | 0.7474 | 2.2576 | 0.4377 | 0.1346 | 1.4235 | 1.6848 | 0.4054 | 7.0021 |
| 63 | 68 | 4.7980 | 1.5586 | 14.7704 | 1.4287 | 0.8259 | 2.4716 | 0.4153 | 0.1211 | 1.4236 | 2.0165 | 0.5033 | 8.0802 |
| 64 | 68 | 7.8760 | 2.0749 | 29.8959 | 1.4420 | 0.7971 | 2.6085 | 0.3117 | 0.0774 | 1.2546 | 1.2585 | 0.2625 | 6.0331 |
| 65 | 68 | 4.7213 | 1.5286 | 14.5829 | 1.4006 | 0.8078 | 2.4285 | 0.4536 | 0.1383 | 1.4878 | 1.9522 | 0.4914 | 7.7560 |
| 66 | 68 | 5.2042 | 1.6475 | 16.4393 | 1.5297 | 0.8529 | 2.7435 | 0.4256 | 0.1260 | 1.4379 | 1.6671 | 0.4033 | 6.8918 |
| 67 | 68 | 4.7698 | 1.5572 | 14.6101 | 1.4137 | 0.8202 | 2.4365 | 0.4358 | 0.1311 | 1.4489 | 1.8776 | 0.4689 | 7.5188 |
| 68 | 68 | 6.1083 | 1.8042 | 20.6803 | 1.6108 | 0.8983 | 2.8882 | 0.4522 | 0.1360 | 1.5028 | 1.9744 | 0.4796 | 8.1270 |
| 69 | 68 | 4.7383 | 1.5466 | 14.5171 | 1.4122 | 0.8192 | 2.4344 | 0.4383 | 0.1320 | 1.4559 | 1.8792 | 0.4678 | 7.5493 |
| **Combined results** | | 4.7941 | 4.1800 | 5.4983 | 1.4156 | 1.3250 | 1.5125 | 0.4507 | 0.3898 | 0.5210 | 1.9654 | 1.6610 | 2.3256 |

Abbreviations: OR, odds ratio; CI, confidence interval.
